# Accuracy of ECG-based screening for sleep-disordered breathing: a survey of all male workers in a transport company

**DOI:** 10.1007/s11325-012-0681-7

**Published:** 2012-03-20

**Authors:** Junichiro Hayano, Teruomi Tsukahara, Eiichi Watanabe, Fumihiko Sasaki, Kiyohiro Kawai, Hiroki Sakakibara, Itsuo Kodama, Tetsuo Nomiyama, Keisaku Fujimoto

**Affiliations:** 1 Center for Medical Education Research and Development, Nagoya City University Graduate School of Medical Sciences, 1 Kawasumi Mizuho-cho Mizuho-ku, Nagoya, Aichi 467-8601 Japan; 2 Department of Preventive Medicine and Public Health, Shinshu University School of Medicine, 3-1-1 Asahi, Matsumoto, Nagano, 390-8621 Japan; 3 Division of Cardiology, Department of Internal Medicine, Fujita Health University School of Medicine, 1-98 Dengakugakubo, Kutsukake-cho, Toyoake, Aichi 470-1192 Japan; 4 Takaoka Clinic Nagoya, 2-28-24 Izumi, Higashi-ku, Nagoya, Aichi 461-0001 Japan; 5 Suzuken Company Limited, 8 Higasikataha, Higashi-ku, Nagoya, Aichi 461-0015 Japan; 6 Nagoya University, Furo-cho, Chikusa-ku, Nagoya, Aichi 464-8601 Japan; 7 Department of Biomedical Laboratory Sciences, Shinshu University School of Health Sciences, 3-1-1 Asahi, Matsumoto, Nagano, 390-8621 Japan

**Keywords:** Apnea–hypopnea index, Cyclic variation of heart rate, Electrocardiogram, Sleep apnea, Sleep-disordered breathing, Population

## Abstract

**Purpose:**

Sleep-disordered breathing (SDB) is associated with increased risk for cardiovascular morbidity and mortality and for sleepiness-related accidents, but >75 % of the patients remain undiagnosed. We sought to determine the diagnostic accuracy of ECG-based detection of SDB when used for population-based screening.

**Methods:**

All male workers, mostly truck drivers, of a transport company (*n* = 165; age, 43 ± 12 years) underwent standard attended overnight polysomnography. Cyclic variation of heart rate (CVHR), a characteristic pattern of heart rate associated with SDB, was detected from single-lead ECG signals during the polysomnography by a newly developed automated algorithm of autocorrelated wave detection with adaptive threshold (ACAT).

**Results:**

Among 165 subjects, the apnea–hypopnea index (AHI) was ≥5 in 62 (38 %), ≥15 in 26 (16 %), and ≥30 in 16 (10 %). The number of CVHR per hour (CVHR index) closely correlated with AHI [*r* = 0.868 (95 % CI, 0.825–0.901)]. The areas under the receiver operating characteristic curves for detecting subjects with AHI ≥5, ≥15, and ≥30 were 0.796 (95 % CI, 0.727–0.855), 0.974 (0.937–0.993), and 0.997 (0.971–0.999), respectively. With a predetermined criterion of CVHR index ≥15, subjects with AHI ≥15 were identified with 88 % sensitivity and 97 % specificity (likelihood ratios for positive and negative test, 30.7 and 0.12). The classification performance was retained in subgroups of subjects with obesity, hypertension, diabetes mellitus, dyslipidemia, and decreased autonomic function.

**Conclusions:**

The CVHR obtained by the ACAT algorithm may provide a useful marker for screening for moderate-to-severe SDB among apparently healthy male workers.

## Introduction

Sleep-disordered breathing (SDB) is a contemporary challenge to health and well-being [1–3]. Studies have demonstrated that SDB increases the risk of hypertension [4], coronary artery disease [5, 6], stroke [7], diabetes [8], chronic kidney disease [9], depression [10], cognitive impairment, diminished quality of life [11], and motor vehicle crashes [12]. Despite these facts and the availability of effective treatments, at least 75 % of the patients with SDB remain undiagnosed [13]. Establishing efficient medical and public health system for SDB screening is therefore an urgent concern.

ECG seems potentially the most practical tool for screening for SDB. Episodes of SDB are accompanied by a characteristic pattern of heart rate, known as cyclic variation of heart rate (CVHR) [14], which consists of bradycardia during apnea followed by rapid return with its cessation. Earlier studies have reported several ECG-based algorithms for detecting CVHR that demonstrate good classification performance between SDB patients and normal subjects [15–18]. These studies, however, were based on observations either in small test data [15, 16] or in subjects referred for polysomnography with suspected SDB [18–20]. The classification performance depends on pretest probability [21] and comorbidities that are known to affect CVHR [14, 22].

In the present study, we therefore sought to determine the diagnostic accuracy of ECG-based SDB detection in population-based screening. We used a newly developed automated algorithm of autocorrelated wave detection with adaptive threshold (ACAT) for detecting CVHR [19]. This algorithm has been reported to identify patients with moderate-to-severe SDB with 83 % sensitivity and 88 % specificity among 862 patients referred for diagnostic polysomnography [19]. We performed overnight polysomnography in all male workers in a transport company and examined the classification performance of the algorithm.

## Methods

### Participants

All male workers of a transport company were eligible for inclusion in this study. Most of them were involved in driving trucks for long distances. All male workers gave their written informed consent to participate in this study. Consequently, we performed a complete survey of all male workers (*n* = 165) of this company. All subjects were racially Japanese.

### Protocol

The protocol of this study was approved by the Human Ethics Committee of Shinshu University School of Medicine (No. 658, January 4, 2006). Between February 2006 and August 2007, subjects underwent polysomnography as well as a medical checkup for medical history, physical examination, blood sampling, and daytime sleepiness by the Epworth Sleepiness Scale.

### Sleep study

Standard overnight attended polysomnography was performed in a university laboratory starting at 20:00 h, and the data were collected from 21:00 h to 06:00 h the next morning. The bed was covered with a sheet-form respiratory movement sensor for the purpose of another study [23]. The polysomnogram was recorded with a digital polygraph (Alice III; Chest Co. Ltd., Tokyo, Japan). We used the standard polysomnographic montages consisting of C4-A2, C3-A1, O2-A1, and O1-A2 electroencephalograms, left and right electrooculograms, a submental electromyogram, a nasal pressure cannula, oronasal airflows, left and right tibial electromyograms, thoracoabdominal inductance plethysmograms, pulse oximetric arterial blood oxygen saturation (SpO_2_), a neck microphone, body position sensors, and a modified lead II ECG.

The sleep stages and respiratory events were scored according to the AASM Manual for the Scoring of Sleep and Associated Events [24]. The average hourly frequency of apneic and hypopneic episodes was defined as apnea–hypopnea index (AHI). Total sleep time (TST) was used as the denominator in the calculation of AHI. Subjects with AHI between 5 and 15 were defined as mild SDB, those with AHI between 15 and 30 as moderate SDB, and those with AHI ≥30 as severe SDB.

### Detection of CVHR

**Fig. 1:**
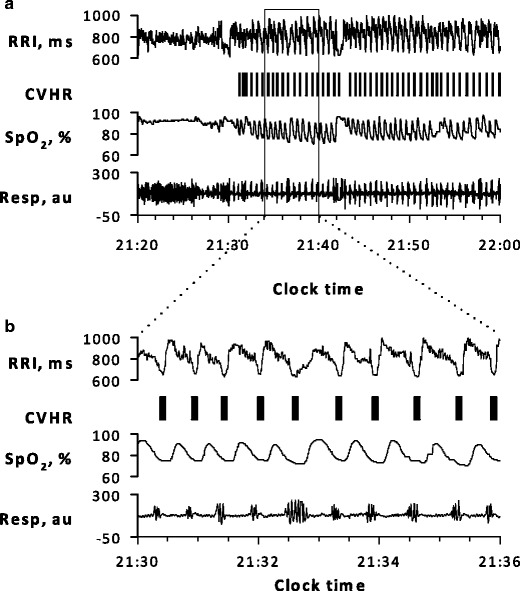
**a** A strip of polysomnographic data with the markers of cyclic variation of heart rate (CVHR) detected by the autocorrelated wave detection with adaptive threshold (ACAT) algorithm in a representative subject with sleep-disordered breathing (SDB). The temporal positions of detected CVHR are indicated with *short vertical bars*. **b** Closer view of the data in the *open box* in (**a**). The ACAT algorithm detected the nadirs of cyclic dips in interbeat intervals that accompany apnea–hypopnea events. *RRI* interbeat interval measure as R–R interval of ECG, *Resp* respiration by oronasal airflow, *SpO*_*2*_ pulse oximetric arterial blood oxygen saturation

The modified lead II ECG signal (sampling frequency, 100 Hz) for the entire length of each polysomnogram was extracted. All QRS complexes were identified and labeled as normal (sinus rhythm), ventricular ectopic, supraventricular ectopic, and artifact, and R–R interval time series were generated using only normal-to-normal intervals. The detection of CVHR was performed by a technician who was blind to both subject characteristics and other polysomnographic findings. Using the automated ACAT algorithm [19], CVHR was detected as the cyclic occurrence of autocorrelated waves (Fig. 1). See Appendix for the detail of the ACAT algorithm.

We calculated CVHR index as the average number of CVHRs (dips meeting the criteria) per hour of time in bed (TIB). We used CVHR index ≥15 that had been determined by a previous study [19] as the criterion for identifying patients with moderate-to-severe SDB. We also calculated standard deviation of normal-to-normal R–R intervals (SDNN) as an index of cardiac autonomic function and defined subjects with a SDNN <75 ms (mean minus 1 SD) as having decreased autonomic function.

### Statistical analysis

The correlation and agreement between the CVHR index and AHI were evaluated by Pearson’s product moment correlation coefficient and the limits of agreement of Bland and Altman [25], respectively. The classification performance of the CVHR index was evaluated by receiver operating characteristic (ROC) curve analysis. The difference in the performance was evaluated by comparing the areas under the curve (AUC) by the method of Hanley and McNeil [26]. The guideline of the American College of Chest Physicians and other related academic societies [27] recommends the use of likelihood ratios (LRs) for assessing the utility of diagnostic alternatives to polysomnography; it also offers multiple threshold approaches, one for best reducing the probability (false negative rate) and the other for best increasing the probability (true positive rate), allowing a *gray zone*, where the result of screening test is accepted as *indeterminate*. Accordingly, we calculated LRs for positive test (LR+) and negative test (LR−) to evaluate the classification performance and also performed interval LR analysis to determine the thresholds for increasing and reducing the post-test probabilities. We assessed the changes in disease probability with the levels of LR as follows: <0.05, very large reduction; 0.05–0.1, large reduction; 0.1–0.2, modest reduction; 0.21–5.0, little change; 5.1–10.0, modest increase; 10.1–20.0, large increase; and >20.0, very large increase. Data are presented as mean ± SD (range) when appropriate. We defined a *P* value <0.05 as statistically significant.

## Results

Table 1 summarizes the characteristics of the subjects. They included 57 (35 %) patients with hypertension, 13 (8 %) with diabetes mellitus, and 83 (50 %) with dyslipidemia. The polysomnography revealed that the sleep efficiency was 78 ± 13 % and that the median (interquartile rage) of AHI was 3.1 (0.9–8.6). Out of 165 subjects, 62 (38 %) had AHI ≥5, including 36 (22 %) with mild, 10 (6 %) with moderate, and 16 (10 %) with severe SDB.

**Table 1:** Characteristics of the study subjects

*N*	165
Age, years	43 ± 12 (18–69)
Height, cm	170 ± 6 (155–186)
Weight, kg	68 ± 11 (49–112)
BMI, kg/m^2^	24 ± 3 (16–37)
BMI ≥25 kg/m^2^	50 (30 %)
BMI ≥35 kg/m^2^	1 (1 %)
Systolic blood pressure, mmHg	126 ± 18 (96–186)
Diastolic blood pressure, mmHg	81 ± 12 (56–118)
ESS score	5 ± 4 (0–19)
ESS score >11	11 (7 %)
ESS score >16	3 (2 %)
Comorbidity	
Hypertension, *n* (%)	57 (35 %)
Diabetes mellitus, *n* (%)	13 (8 %)
Dyslipidemia, *n* (%)	83 (50 %)
Polysomnographic features	
TIB, min	623 ± 87 (276–770)
TST, min	485 ± 104 (125–683)
Rapid-eye-movement period, min	82 ± 35 (8–180)
Sleep efficiency, %	78 ± 13 (27–98)
AHI (TIB), events/h	8 ± 13 (0–63)
AHI (TST), events/h	9 ± 14 (0–67)
Maximum O_2_ desaturation, %	9 ± 6 (0–35)
SDNN, ms	105 ± 32 (44–179)

Data are shown as number (%) for categorical variables and mean ± SD (range) for continuous variables

*AHI* apnea–hypopnea index, *BMI* body mass index, *ESS* Epworth Sleepiness Scale, *SDNN* standard deviation of normal-to-normal R–R interval, *TIB* time in bed, *TST* total sleep time

The ECG in all 165 subjects showed sinus rhythm. The ratio of analyzable R–R intervals to the recording length was 94 ± 9 % (44–100 %). Although this ratio was <70 % only in four subjects due to recording noises, these subjects were also included in the analysis (intension-to-diagnose policy). Both the AHI and the CVHR index were <15 in all of these subjects but one, in whom the ratio of analyzable R–R intervals was only 44 % and the CVHR index and AHI were 17.6 and 35.8, respectively.

**Fig. 2:**
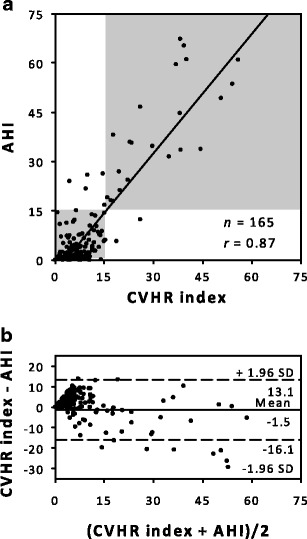
Scatter graphs with the regression line (**a**) and Bland and Altman plot (**b**) for the relationships between the CVHR index and apnea–hypopnea index (*AHI*). In (**b**), the *horizontal solid line* and *dashed lines* indicate the mean difference and the upper and lower limits of agreement (mean ± 1.96 SD), respectively

**Fig. 3:**
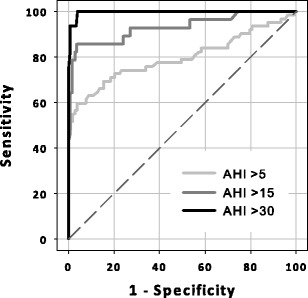
Receiver operating characteristic (ROC) curves of the CVHR index to identify patients with different severity of SDB

Figure 2 shows the relationship and agreement between the CVHR index and AHI. The CVHR index was correlated with AHI [*r* = 0.868 (95 % CI, 0.825–0.901)]. The Bland and Altman plot showed upper and lower limits of agreement of 13.1 and –16.1, respectively, with a tendency of increasing difference with increasing AHI. The ROC curve analysis for identification of patients with moderate-to-severe SDB showed an AUC of 0.974 (SE, 0.0142; *P* <0.0001) and by the criterion of CVHR index ≥15, the patients were detected at 88 % sensitivity and 97 % specificity (Table 2). A good classification performance was also observed in detecting severe SDB [AUC (SE), 0.997 (0.003), *P* < 0.0001] with the optimal cutoff criterion of CVHR index ≥17, although the performance in detecting mild SDB was modest (Table 2 and Fig. 3).

**Table 2:** Classification performance of the CVHR index for identifying patients with different severity of SDB

	*n*	ROC curve AUC (SE)	CVHR index criterion	Sensitivity	Specificity	PPV	NPV	LR+	LR−
AHI ≥5	62	0.796 (0.0414)*^a^	≥12^c^	60 %	96 %	90 %	80 %	15.4	0.42
AHI ≥15	26	0.974 (0.0142)*	≥14^c^	92 %	96 %	83 %	99 %	25.7	0.08
	–	–	≥15^b^	88 %	97 %	85 %	98 %	30.7	0.12
AHI ≥30	16	0.997 (0.003)*	≥17^c^	100 %	96 %	72 %	100 %	24.8	0.00

*AUC* area under the curve, *CVHR* cyclic variation of heart rate, *LR +* likelihood ratio for positive test, *LR* − likelihood ratio for negative test, *NPA* negative predictive value, *PPV* positive predictive value, *ROC* receiver operating characteristic

*P < 0.0001 (against AUC = 0.5)

^a^Significantly lower than AUCs for AHI ≥15 and AHI ≥30

^b^Predetermined criterion of CVHR index for detecting moderate-to-severe SDB [19]

^c^Cutoff threshold determined in the present study as the value corresponding to the highest average of sensitivity and specificity

**Table 3:** Interval likelihood ratio of the CVHR index for identifying patients with different severity of SDB

	CVHR index				Pretest probability
	<10 (*n* = 110)	10–15 (*n* = 29)	15–30 (*n* = 15)	≥30 (*n* = 11)	
AHI ≥5	0.39 (19 %)	1.78 (52 %)	∞ (100 %)	∞ (100 %)	38 %
AHI ≥15	0.10 (2 %)	0.40 (7 %)	14.7 (73 %)	∞ (100 %)	16 %
AHI ≥30	0.00 (0 %)	0.00 (0 %)	4.66 (33 %)	∞ (100 %)	10 %

Data are LR (post-test probability, %)

*CVHR* cyclic variation of heart rate, *LR* likelihood ratio

**Table 4:** Effects of age and comorbidities on the classification performance of the CVHR index (cutoff, ≥15)

Subgroup	*n*	AHI ≥15 *n* (%)	ROC curve AUC (SE)	Sensitivity	Specificity	PPV	NPV	LR+	LR−
Age <52 years	124	12 (10 %)	0.963 (0.0225)*	83 %	97 %	77 %	98 %	31.1	0.17
Age ≥52 years	41	14 (34 %)	0.995 (0.0064)*	93 %	93 %	93 %	93 %	12.5	0.08
BMI <25 kg/m^2^	115	9 (8 %)	0.968 (0.0266)*	89 %	98 %	80 %	99 %	47.1	0.11
BMI ≥25 kg/m^2^	50	17 (34 %)	0.970 (0.0223)*	898 %	97 %	94 %	94 %	29.1	0.12
Hypertension (−)	108	7 (6 %)	0.996 (0.0040)*	100 %	99 %	88 %	100 %	101.0	0.00
Hypertension (+)	57	19 (33 %)	0.939 (0.0369)*	89 %	95 %	88 %	93 %	167.0	0.11
Diabetes (−)	152	20 (13 %)	0.952 (0.0567)*	95 %	96 %	79 %	99 %	25.8	0.05
Diabetes (+)	13	6 (46 %)	0.952 (0.0567)*	83 %	100 %	100 %	88 %	∞	0.17
Dyslipidemia (−)	82	5 (6 %)	0.990 (0.0095)*	80 %	99 %	80 %	99 %	61.6	0.20
Dyslipidemia (+)	83	21 (25 %)	0.970 (0.0191)*	90 %	97 %	91 %	97 %	28.0	0.10
SDNN ≥75 ms	139	20 (14 %)	0.982 (0.0144)*	95 %	97 %	83 %	99 %	28.3	0.05
SDNN <75 ms	26	6 (23 %)	0.958 (0.0368)*	83 %	95 %	83 %	95 %	16.7	0.18

Abbreviations are explained in the footnote to Table 2

*P < 0.0001 (against AUC = 0.5)

Table 3 shows the results of interval LR analysis. There was a very large reduction in the probability of severe SDB (AHI ≥30), when CVHR index was <15; a very large increase in the probability of SDB (AHI ≥5) and a large increase in that of moderate-to-severe SDB (AHI ≥15), when CVHR index was ≥15; and a very large increase in the probability of moderate-to-severe SDB (AHI ≥30), when CVHR index was ≥30.

Age, comorbidities, and autonomic function had no substantial impact on the classification performance in detecting moderate-to-severe SDB by the criterion of CVHR index ≥15 (Table  4). The AUC of the ROC curve did not differ significantly between subjects grouped by older age (≥52 years, the 75th percentile value) or by the presence of obesity (BMI ≥25 kg/m^2^), hypertension, diabetes mellitus, dyslipidemia, and decreased cardiac autonomic function.

## Discussion

This is the first population-based study to report the diagnostic accuracy of automated ECG-based detection of SDB. In a survey of all male workers of a transport company, the ACAT algorithm identified moderate-to-severe SDB patients with 88 % sensitivity and 97 % specificity with a predetermined criterion of CVHR index ≥15. Also, interval LR analysis revealed that CVHR index <15 is a useful criterion for reducing the probability of severe SDB (AHI ≥ 30), CVHR index ≥15 is for increasing the probability of SDB (AHI ≥ 5), and CVHR index ≥30 is for increasing the probability of moderate-to-severe SDB (AHI ≥ 15). The accuracy was retained in the subgroups divided by age, obesity, hypertension, diabetes mellitus, dyslipidemia, and decreased autonomic function. These observations indicate that the automated ECG-based detection of CVHR during sleep may be used as a screening tool for moderate-to-severe SDB among male workers.

This study has several strengths as a diagnostic study [28]. First, we could performed a survey of all male workers in a company and obtain both the index test (CVHR index) and the reference standard (AHI by a standard overnight attended polysomnography) from all subjects. The observations were of complete samples from a population of male workers. Second, because both CVHR index and AHI were evaluated simultaneously, there was neither time lag nor treatment effects between the index test and the reference standard. Third, the majority of the subjects consisted of long-distance truck drivers, a representative population in whom the screening for SDB is of particular importance [12]. Finally, we estimated the variability of diagnostic accuracy between subgroups divided by age, obesity, and the diseases commonly observed among SDB patients [3].

The ACAT algorithm may perform excellently in comparison to other ECG-based algorithms for SDB detection. Several earlier studies [15–17] have used the Physionet Sleep Apnea–ECG database (http://www.physionet.org/physiobank/database/apnea-ecg/) to examine the performance of the algorithms and reported an accuracy of 90–100 %. This database consists of 70 ECG samples including 42 patients with AHI ≥15 and 23 normal subjects (AHI < 5). The ACAT algorithm also showed a good performance [AUC, 0.979 (95 % CI, 0.912–0.998); 90 % sensitivity and 100 % specificity] for this database [19]. Although only a few studies have examined the performance of the algorithms in clinical settings, they have reported modest diagnostic accuracies [18, 20, 29]. In a study of 150 patients referred to a university hospital for clinically suspected SDB, Roche et al. [18] reported that an algorithm that used the relative power of the very low frequency component detected patients with AHI ≥15 with an AUC of 0.70, a sensitivity of 64 %, and a specificity of 69 %. The ACAT algorithm, however, has been reported to detect patients with AHI ≥15 with an AUC of 0.913, a sensitivity of 83 %, and specificity of 88 % in a study of 887 consecutive patients referred for diagnostic polysomnography [19].

The advantage of the ACAT algorithm may be derived from its feature of an adaptive threshold. By visual inspection of R–R intervals, Guilleminault et al. [14] reported that the CVHR was not observed in patients with denervated transplanted heart or with severe autonomic neuropathy and that it was blunted in those with moderate autonomic neuropathy and with Shy–Drager syndrome. They also observed in subjects with normal autonomic function that the bradycardic component of the CVHR showed a progressive reduction with increasing doses of intravenous atropine. Cardiac vagal activity decreases with aging [30] and is reduced in patients with diabetes mellitus [31] and dyslipidemia [32]. These conditions are commonly associated with SDB [4, 8, 33] and have been reported to increase the failure of CVHR detection [22]. The adaptive threshold of the ACAT algorithm may help maintain the good classification performance even in these subjects.

The ACAT algorithm requires only R–R interval time series data. The most promising application of this algorithm seems to be screening for sleep apnea by Holter ECG. According to the *Survey of Medical Care Activities in Public Health Insurance* conducted by the Japanese Ministry of Health, Labor and Welfare in 2008, at least 1,270,000 Holter ECG examinations are performed each year in Japan alone. Holter ECG is used as a routine examination in most clinical facilities; in many of them, digitized Holter ECG data are stored in re-analyzable forms. Also, Holter ECG scanners used for analyzing recorded ECG signals unexceptionally measure beat-to-beat R–R intervals as a fundamental function. Thus, the data that are necessary for the ACAT algorithm can be obtained at small additional cost. Furthermore, the cardiovascular patients, the most likely subjects of Holter ECG examination, are also the high-risk population of SDB [3]. The installation of ACAT algorithm into Holter ECG scanners may provide useful screening tool for sleep apnea in these patients.

The present study has several limitations. First, the studied population consisted of only male workers. Thus, the performance of the ACAT algorithm in other populations is unclear, although our previous observation in subjects referred for polysomnography indicated that the classification performance was maintained even in 155 female subjects and in 145 subjects aged ≥65 years [19]. Second, we studied the ECG signal extracted from polysomnographic recordings obtained in a laboratory. The performance may be affected by the recording environment. It is not clear if our findings can be directly extended to ambulatory ECG recorded during daily life. Third, we used TIB as the denominator for calculating CVHR index, while we used TST as the denominator for AHI. This might have affected the association between CVHR and AHI. Their correlation coefficients were comparable, however, even when TIB was used for calculating AHI (*r* = 0.87 vs. 0.86). Fourth, we did not evaluate the effects of medications because we were unable to have consent from the subjects to access their clinical medical recordings. We performed, however, a full health checkup of the subjects and observed that the classification performance was retained even in the subjects with hypertension, diabetes mellitus, dyslipidemia, and decreased autonomic function. Fifth, it should be noted that the CVHR index may not be used for estimating AHI. Although the CVHR index correlated with AHI, the Bland and Altman plot showed increasing disagreement with increasing AHI. Also, the optimal cutoff criterion of CVHR index for identifying patients with an AHI ≥30 was ≥17. These results suggest that the CVHR index, if used as a quantitative index, would underestimate the AHI in patients with severe SDB. The CVHR index should not be used for AHI estimation; it should be used only for per-subject screening tool for moderate-to-severe SDB. Finally, to confirm the cost-effectiveness of ECG screening for sleep apnea, comparisons with other portable monitors such as those with oximetry are necessary in future studies.

## Conclusions

We observed that analysis of CVHR from ECG by the automated algorithm of ACAT identified patients with moderate-to-severe SDB in a survey of all male workers in a transport company. Our observations suggest that automated ECG detection of CVHR may be a useful screening tool for moderate-to-severe SDB among male workers.

## Figures and Tables

**Fig. 4:**
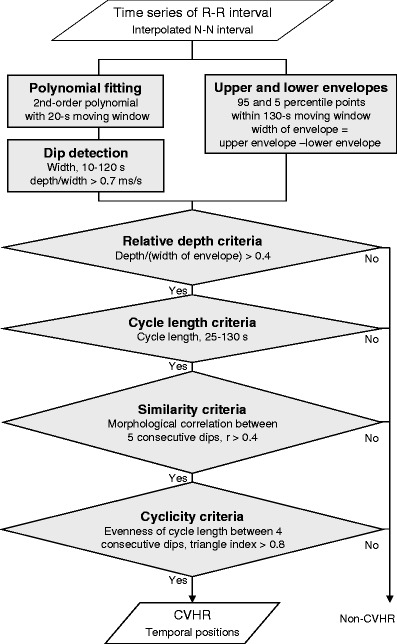
Algorithm of autocorrelated wave detection with adaptive threshold (ACAT). The algorithm detects the temporal positions of cyclic variation of heart rate (CVHR) in the interbeat interval time series as the cyclic and autocorrelated dips that meet four specific criteria (modified from Fig. 1 in Ref. [19])

**Fig. 5:**
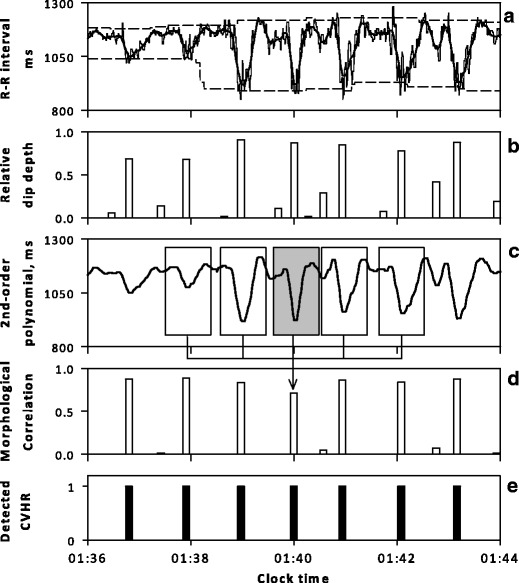
Schema showing the processes for detecting CVHR by the ACAT algorithm. **a** Original R–R interval (*thin solid line*), second-order polynomial fitting line (*heavy solid line*), and the upper and lower envelopes of the fitting line (*dashed lines*). **b** The depth of dips relative to the instantaneous width of envelopes. **c** The morphological comparisons of each dip (*shaded box*) with the two preceding and two subsequent dips (*open boxes*). **d** Mean morphological correlation coefficients. **e** Temporal positions of dips detected as CVHR

## Appendix

Automated algorithm of autocorrelated wave detection with adaptive threshold (ACAT) for detecting cyclic variation of heart rate (CVHR) [19]

The ACAT algorithm is a time-domain method that uses only interbeat interval data. The algorithm detects the CVHR as cyclic and autocorrelated dips in interbeat interval time series and determines the temporal position of the individual dips comprising the CVHR (Figs. 4 and 5). The processes of the ACAT algorithm were as follows: Interbeat interval time series were smoothed by second-order polynomial fitting, and all dips in the smoothed trend with widths between 10 and 120 s and depth-to-width ratios of >0.7 ms/s were detected. Also, the upper and lower envelopes of the interbeat interval variations were calculated as the 95th and 5th percentile points, respectively, within a sifting window with a width of 130 s. Then, the dips that met the following criteria were considered CVHR: (1) a relative dip depth >40 % of the envelope range at that point (adaptive threshold), (2) interdip intervals (cycle length) between 25 and 130 s, (3) a waveform similar to those of the two preceding and two subsequent dips with a mean morphological correlation coefficients >0.4 (autocorrelated wave), and (4) three cycle lengths between four consecutive dips that meet the following equivalence criteria:

$$ \left( {{3} - {2}\;{l_{{1}}}/s} \right)\left( {{3} - {2\;}{l_{{2}}}/s} \right)\left( {{3} - {2}\;{l_{{3}}}/s} \right) > 0.{8}, $$

where *l*_1_, *l*_2_, and *l*_3_ are three consecutive cycle lengths and *s* = (*l*_1_ + *l*_2_ + *l*_3_)/3. The number of dips comprising the CVHR is counted and the mean number of the dips per hour of time in bed is calculated as the CVHR index.
